# Toward Precision Medicine: Exploring the Landscape of Biomarkers in Acute Kidney Injury

**DOI:** 10.3390/biom14010082

**Published:** 2024-01-08

**Authors:** Nicole Nourie, Rita Ghaleb, Carmen Lefaucheur, Kevin Louis

**Affiliations:** 1 Department of Nephrology and Kidney Transplantation, Saint Louis Hospital, Assistance Publique-Hôpitaux de Paris, 75010 Paris, France; 2 Human Immunology and Immunopathology, Inserm UMR 976, Université Paris Cité, 75010 Paris, France; 3 Faculty of Medicine, Saint Joseph University, Beirut 1104 2020, Lebanon

**Keywords:** acute kidney injury, biomarkers, chronic kidney disease

## Abstract

Acute kidney injury (AKI) remains a complex challenge with diverse underlying pathological mechanisms and etiologies. Current detection methods predominantly rely on serum creatinine, which exhibits substantial limitations in specificity and poses the issue of late-stage detection of kidney injury. In this review, we propose an up-to-date and comprehensive summary of advancements that identified novel biomarker candidates in blood and urine and ideal criteria for AKI biomarkers such as renal injury specificity, mechanistic insight, prognostic capacity, and affordability. Recently identified biomarkers not only indicate injury location but also offer valuable insights into a range of pathological processes, encompassing reduced glomerular filtration rate, tubular function, inflammation, and adaptive response to injury. The clinical applications of AKI biomarkers are becoming extensive and serving as relevant tools in distinguishing acute tubular necrosis from other acute renal conditions. Also, these biomarkers can offer significant insights into the risk of progression to chronic kidney disease CKD and in the context of kidney transplantation. Integration of these biomarkers into clinical practice has the potential to improve early diagnosis of AKI and revolutionize the design of clinical trials, offering valuable endpoints for therapeutic interventions and enhancing patient care and outcomes.

## 1. Introduction

Currently, international guidelines for the diagnosis and stratification of acute kidney injury (AKI) are based solely on the loss of glomerular function, assessed through serum creatinine (SCr) dosage and urinary output. This has been the norm for more than 100 years. Although these guidelines and classifications, such as RIFLE, AKIN, and KDIGO 2012, are essential in defining, stratifying, and treating patients, it has become evident that they primarily focus on renal function and excretion, lacking precision regarding the underlying injury mechanism. Indeed, it has become apparent that a phenomenon like acute kidney injury involving multiple aspects, such as etiology, pathophysiological mechanisms, prognosis, and treatment, should not be reduced into a single biochemical test. SCr might not be the ideal biomarker for acute kidney injury for multiple reasons. Firstly, creatinine production is not always the reflection of a stable state; it is the product of the metabolism of creatine and phosphocreatine in muscle cells. This production can vary significantly with factors such as age, gender, muscle mass, and protein intake. This variability also underscores that creatinine requires time to accumulate, rendering it a delayed marker. Furthermore, it is not specific to renal or tubular injury; elevated creatinine levels can be observed in all phenotypes ranging from prerenal azotemia to obstructive disease. Therefore, its elevation does not provide substantial evidence regarding the location, etiology, or treatment of the injury. Finding the ideal biomarker can be challenging, especially in the context of AKI. It should not only reflect the renal injury specificity and offer mechanistic insight but also be reproducible, hold a prognostic capacity, and be affordable and noninvasive.

Biomarkers are defined and categorized according to the BEST (Biomarkers, EndpointS, and other Tools) resource, a joint task force between the Food and Drug Administration (FDA) and National Institutes of Health (NIH), which divides biomarkers into different categories portraying their functions ranging from diagnosis and monitoring to prognosis and predictive value, leading to clinical and surrogate endpoints biomarker [1]. Ideally, the biomarker should then undergo verification in multicenter cohorts, followed by prospective cohorts focusing on specific outcomes. After these validation steps, the biomarker should also be tested in specifically designed clinical trials to ensure its role in changing patient outcomes in clinical practice [2]. Finally, in the case of AKI, an ideal biomarker may not replace SCr and urine output but should rather be considered as of additional value, as illustrated by an insightful model proposed by De Oliveira et al. [3]. In this model, the three elements are combined in a dynamic visualization of the actual insult, combining injury with dysfunction. This concept brings us closer to the truth, where etiologies may range from initial phases of damage, where the injury is represented by an increase in biomarkers while function is still preserved, indicated by normal serum creatinine and urine output. On the other end of the spectrum, we find etiologies with dysfunction, leading to a rise in creatinine and a decrease in urine output, but without injury or an increase in biomarkers, as seen in reversible volume depletion. In this review, we present a comprehensive summary of AKI biomarkers, along with their clinical applications, prognostic value, and their potential for improving risk stratification and therapeutic guidance.

## 2. Biomarkers Uncovered: From Secretion to Significance

**Figure 1:**
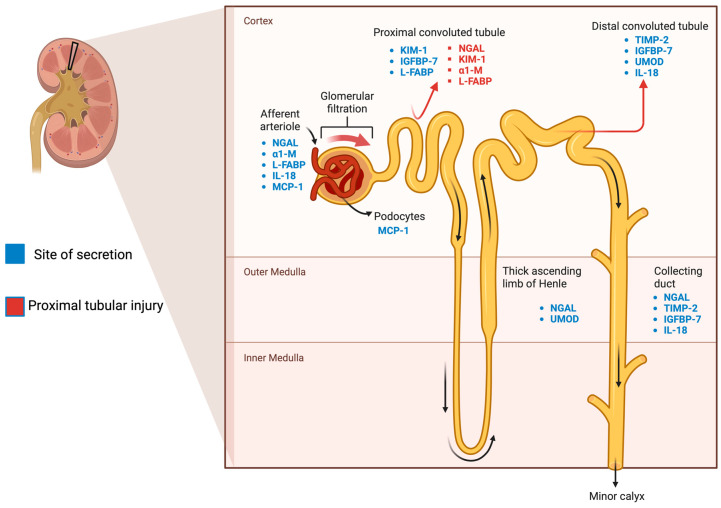
Distribution of AKI biomarkers across the tubule. Biomarkers’ site of secretions before renal excretion (visualized by the arrows throughout the tubules) or systemic release. Locations include endothelial cells, tubular epithelial cells, interstitial cells, and podocytes.

In approaching AKI biomarkers, it is essential to recognize that various categorization methods exist, including differentiation by site of secretion or whether these are assessed in blood or urine (Figure 1). However, it seems more relevant to categorize biomarkers according to their alignment with underlying pathological processes. Here, we propose a classification into four categories: tubular injury, tubular function, kidney inflammation, and the adaptive response to injury encompassing repair and fibrosis.

### 2.1. Tubular Injury

#### 2.1.1. NGAL, Neutrophil Gelatinase-Associated Lipocalin

NGAL, also known as lipocalin 2 (LCN2), plays a role in facilitating the transport of hydrophilic substances across cell membranes [4], participates in cellular defense mechanisms, and influences iron metabolism [5]. NGAL is not exclusively secreted by the kidney but is found in epithelial cells throughout various organs, such as the lungs and the digestive system, in response to various inflammatory signals [6]. The renal secretion of NGAL, particularly in the ascending limb of the loop of Henle and within the collecting ducts, accounts for a negligible fraction of the overall plasma NGAL concentration, as confirmed by renal vein sampling [7]. Plasma NGAL is completely reabsorbed by the proximal tubule after it is freely filtered. Therefore, the detection of urinary NGAL of plasma origin is only possible in the case of proximal tubular injury or when the synthesis exceeds the tubular reabsorption capacity. On the other hand, a decrease in GFR leads to a decrease in NGAL excretion and, therefore, increases its plasma accumulation. While both plasma and urinary NGAL concentrations have been used as biomarkers of tubular injury, considering the factors mentioned above, urinary NGAL appears to be a more specific indicator for kidney injury. It is one of the earliest biomarkers to be detected following injury, as demonstrated in a rat model of renal ischemia-reperfusion injury. In this model, urinary NGAL was detected as early as two hours after the injury, with the first urinary output following renal artery occlusion [8].

#### 2.1.2. KIM-1, Kidney Injury Molecule 1

KIM-1, a transmembrane glycoprotein serving as an adhesion molecule, was originally identified in the S3 segment of the proximal tubule. While its expression remains minimal in normal kidneys, it experiences significant upregulation in response to ischemic-reperfusion injury in rat models [9]. This observation was extended to human subjects, where the presence of the soluble released form of urinary KIM-1 was confirmed to be associated with acute tubular necrosis, thus establishing it as a marker of proximal tubular injury [10]. It is worth noting that the genes that code for KIM-1, as well as NGAL, were found to be significantly upregulated late after the injury. This could potentially function as an indicator of persistent renal damage following the initial acute insult and may, therefore, serve as a prognostic factor for the development of subsequent chronic kidney disease (CKD) [11].

#### 2.1.3. TIMP-2 Tissue Inhibitor of Metalloproteinases-2 and IGFBP-7 Insulin-Like Growth Factor-Binding Protein 7

G1 cell cycle arrest is a recognized response mechanism for kidney injury [12]. TIMP-2 and IGFBP-7 play important roles by upregulating the expression of p27 and p53/21, respectively, and consequently inhibiting the progression of the cell cycle through the inhibition of cyclin-dependent protein kinase complexes. Notably, a predominant localization of expression is observed in the area of the collecting duct/distal tubule for TIMP-2 and in the proximal tubules, the collecting duct/distal tubules, and the podocytes for IGFBP7 [13]. The products of urinary TIMP-2 and IGFBP-7 have been established as early biomarkers for acute kidney injury and validated in independent multicenter cohorts [14]. They are currently available commercially under the name NEPHROCHECK. Both biomarkers are continuously expressed in tubular cells, and they are subsequently released and detectable in urine as early as 4 h following kidney injury. This release is more pronounced due to the reduced endocytic uptake by damaged proximal tubules [15].

### 2.2. Tubular Function

#### 2.2.1. Cystatin C

Plasma cystatin C is a well-established biomarker for evaluating glomerular function and filtration, offering an advantage over creatinine because it remains unaffected by variables such as age, gender, muscle mass, and dietary choices. Its production is constant through all nucleated cells, and it is freely filtered in the kidneys without it being secreted or absorbed. Longtime used in clinical practice, leading recently to the development of a cystatin C-based equation, where sex and race variables are not required for the estimation of GFR [16]. Considering that approximately 99% of cystatin C is reabsorbed and metabolized in the proximal tubule following filtration, resulting in its absence in urine under normal physiological conditions, urinary cystatin C additionally serves as a biomarker for tubular function, with a specific focus on proximal tubular function.

#### 2.2.2. α1-M, α1-Microglobuline

In the assessment of tubular function, we also have α1-M, also called protein HC and α_1_-microglycoprotein. Like NGAL, it belongs to the lipocalin family. This glycoprotein, with immunosuppressive proprieties, is synthesized by the liver and released into the bloodstream, where it exists either in a free form or associated with IgA immunoglobulin. The former is freely filtered by the glomerulus and is then mostly reabsorbed by the proximal tubule, making it a marker of proximal tubule marker [17,18].

#### 2.2.3. L-FABP, Liver Type Fatty Acid-Binding Protein

FABP, a member of the superfamily of lipid-binding protein LBP, includes L-FABP, which is secreted by the liver. Their primary role is to bind to fatty acids, particularly in the context of hypoxic injury, facilitating their transport and, therefore, serving as ischemic markers. This function also contributes to the reduction of oxidative stress [19]. Furthermore, L-FABP, secreted by the liver, is also expressed in the kidney, predominantly among the proximal tubular cells. This expression increases during acute proximal ischemic injury, making urinary L-FABP an early marker of tubular function. In an animal model, this increase in L-FABP expression was demonstrated to have a protective effect, reducing the consequences of ischemia by decreasing oxidative stress [20]. Considering its expression in the liver, it is less specific for tubular injury when concurrent chronic liver conditions or sepsis are present. However, research studies have indicated that urinary levels of L-FABP do not correlate proportionally with the increase in their plasma levels but rather with the proximal tubular shedding [21].

#### 2.2.4. UMOD, Uromodulin

Also known as Tamm–Horsfall protein, UMOD is a glycoprotein exclusively secreted in the kidney and specifically by the thick ascending limb of the loop of Henle and the early distal convoluted tubule. It plays a role in salt and blood pressure homeostasis as well as immunomodulatory and anti-inflammatory effects [22]. Diminished urine uromodulin levels have been linked to more pronounced histological lesions, including interstitial fibrosis, tubular atrophy IFTA, and glomerulosclerosis, whereas it was negatively correlated with inflammatory biomarkers such as IL-6, TNFα, and NGAL [23].

### 2.3. Kidney Inflammation

#### 2.3.1. IL-18

IL-18 is a pro-inflammatory cytokine, a member of the IL-1 family produced by kidney macrophages [24,25]. It is found in renal tubular epithelial cells and particularly in the intercalated cells of the distal tubule and the collecting duct [26], but also the proximal tubule [3], possibly reflecting the diverse types and locations of injury and, therefore, the associated inflammatory responses. The levels of IL-18 doubled in animal models of ischemic acute renal failure, while the neutralization of IL-18 before the insult resulted in reduced creatinine level, tubular damage on histology and neutrophile [27], and macrophage infiltration [28], in particular M2 profibrotic macrophages highlighting its role in the progression of AKI to fibrosis and CKD [29].

#### 2.3.2. MCP-1, Monocyte Chemoattractant Protein-1

Serum MCP-1 or Chemokine (CC-motif) ligand 2 (CCL2) is secreted by epithelial, endothelial, and immune cells. Its primary function is to recruit monocytes and macrophages to the site of injury through interaction with the cell surface receptor CCR2 [30]. In the kidney, its expression is triggered by pro-inflammatory cytokines, such as IL-18. When MCP-1 is detected in urine, it stems from local production within the kidney rather than being a product of serum filtration in the glomerulus [31]. MCP-1 is notably recognized for its association with endothelial dysfunction and its role in predicting the activity of lupus nephritis [32].

### 2.4. Adaptive Repair and Fibrosis

#### 2.4.1. EGF, Epidermal Growth Factor

EGF, through its receptor EGFR, plays a role in adaptive repair and fibrosis in acute kidney injury. In a physiological state, away from injury, reduced EGFR activation impairs renal epithelial development. However, when subjected to ischemic or toxic injury in the proximal tubule, EGFR phosphorylation increases. Moreover, experimental deletion of EGFR delays tubular recovery, while its stimulation accelerates the recovery process [33].

#### 2.4.2. Pro and Anti-Angiogenic Markers

VEGF, vascular endothelial growth factor, is essential in angiogenesis and endothelial cell differentiation; its mRNA was mainly detected in podocytes, distal tubules, and collecting ducts [34]. It plays a role in renal repair responses to ischemic injury, where its expression is upregulated in tubular cells. This is followed by the secretion of a free form and mainly extravesicles, promoting endothelial proliferation. Consequently, it helps prevent the progression to CKD in an experimental model of AKI [35]. VEGFR-1 and 2, on the other hand, are expressed on glomerular and peri-tubular endothelial cells; soluble VEGFR-1 is, by contrast, an antiangiogenic molecule that neutralizes the activity of VEGF [36]. Both of these biomarkers were proved effective in predicting AKI in the context of cardiac surgery [37].

## 3. Biomarkers Breakthrough: Exploring Clinical Applications

AKI biomarkers are gradually finding their place in precise clinical applications to assist clinicians in identifying specific populations at risk, guiding therapy, and predicting prognosis and progression to CKD (Table 1). Although they have not yet been incorporated into acute kidney injury (AKI) definitions, several assays are currently available, and significant progress has been made in various areas. In this section, we will explore how the implementation of biomarkers can shift clinical practice towards a more personalized approach. Biomarkers have the potential to identify patients before any loss of function occurs, opening the door to preventive and tailored therapies in AKI. Risk models can also play a crucial role in predicting in-hospital and long-term complications as well as mortality, guiding the development of a personalized follow-up plan shortly after discharge and contributing to long-term risk management. Here, we provide a comprehensive summary of clinical utilization and ongoing randomized clinical trials (Table 2).

### 3.1. Acute kidney Injury Post-Cardiac Surgery

Acute kidney injury occurs in 14% to 30% of hospitalizations for cardiac surgery, worsening the overall prognosis and increasing the risk of mortality, prolonged hospitalization, and healthcare burden. Therefore, the need for early injury detection is crucial. The elevation in SCr or the reduction in urinary output indicates already advanced injury. Translational Research in Biomarker Endpoints Consortium (TRIBE-AKI) was designed to explore the role of postoperative biomarkers in predicting short-term outcomes in the context of cardiac surgery, such as acute kidney injury, the need for dialysis and increased hospital stay [45,58], but also the long-term prognosis, such as overall mortality and progression to CKD.

In 2013, five urinary biomarkers (NGAL, IL-18, KIM-1, L-FABP, and albumin) were measured over the three days following cardiac surgery in 1199 patients from six different centers, with a median follow-up of three years. These biomarkers were found to be independently associated with a 2 to 3.2-fold increase in mortality risk in patients who had clinical AKI and measured for the highest percentile of this panel. In patients without clinical AKI, IL-18 and KIM-1 were also independently correlated with mortality [52]. The concentrations of the five urinary biomarkers were also associated with a longer duration of AKI, and a duration longer than seven days was correlated with a 5-fold increase in three-year mortality [42]. It is important to highlight that hematuria, proteinuria, leucocyte esterase, and nitrite, detected in dipstick tests, can influence the concentration of urinary biomarkers. This impact was evident in a post hoc analysis involving four specific biomarkers (NGAL, IL-8, KIM-1, and L-FABP), where the mentioned factors resulted in false-negative outcomes. In such cases, these biomarkers do not precisely mirror tubular injury [59].

In the same cohort, pre-operative and 3-day post-operative plasma NGAL was measured in 1191 patients undergoing cardiac surgery, with a median follow-up of 3 years. Pre-operative levels of plasma NGAL were also found to be predictive of 3-year mortality after cardiac surgery, indicating prognostic value. Meanwhile, post-operative NGAL lost this association when correlated with SCr, highlighting the different physiopathologies of urinary and plasma NGAL as previously discussed [38].

Later, the consortium investigated perioperative levels of plasma MCP-1 in 972 participants undergoing cardiac surgery. Patients with higher preoperative MCP-1 levels had a higher risk of developing AKI, longer AKI duration, and in-hospital mortality. In this case, preoperative levels were able to predict which patients are at risk of developing AKI and, therefore, guide the physician in stratifying high-risk patients who need earlier preventive measures and interventions [57]. Preoperative urinary α1-M is also added to the list of biomarkers that were found to correlate with a higher risk of post-operative AKI as well as CKD progression and overall mortality [46].

In the same TRIBE-AKI cohort, 1444 adults undergoing cardiac surgery were tested for plasma VEGF and PGF and anti-angiogenic marker VEGFR1 before and within 6 h of the surgery. The assessment revealed that higher postoperative pro-angiogenic markers were associated with a lower incidence and duration of AKI, as well as reduced one-year mortality. Conversely, elevated postoperative VEGFR1 levels were linked to an increased risk of AKI. Notably, the combined angiogenesis panel, incorporating a combination of three of these biomarkers, surpassed the efficacy of individual results. These findings may not be extrapolated to individuals with chronic kidney disease (CKD), where higher levels of VEGF and PGF are respectively correlated with adverse outcomes in diabetic nephropathy and cardiovascular events. This discrepancy mirrors the divergent physiological responses to angiogenesis in acute injury, leading to reparative responses, compared to CKD, which typically results in fibrosis [37].

### 3.2. Cardio-Renal and Hepatorenal Syndromes during Acute Hospital Illness

Identifying the etiology of AKI presents a significant challenge in cirrhotic patients who often present with multiple comorbidities and are susceptible to infectious complications and potential adverse effects from treatments such as diuretics. The etiology of AKI in these cases may range from prerenal azotemia to acute tubular injury (ATI) and hepatorenal syndrome (HRS). Timely and accurate diagnosis is crucial, as AKI significantly increases the risk of mortality in these patients, and management approaches differ significantly.

Although treatment guidelines recommend initiating an intravenous albumin trial at a dose of 1 g/kg of body weight daily for two consecutive days to restore effective arterial blood volume for AKI in cirrhotic patients [60], administrations of large albumin volumes may not always be advantageous and can potentially induce pulmonary edemas. Additionally, the current definition of hepatorenal syndrome relies partly on plasma creatinine, whose interpretation in cirrhosis is limited due to both hepatic insult and a reduction in muscle mass and protein intake. Furthermore, urine excretion of sodium is ineffective in this scenario.

On a therapeutic level, patients with hepatorenal syndrome who had higher levels of creatinine responded less to vasoconstrictors such as Terlipressin in the CONFIRM study, highlighting the need to start this therapy as early as possible [39].

For all these reasons, there is a pressing need to identify valid biomarkers that can aid in distinguishing between the diagnoses of hepatorenal syndrome, tubular injury, or prerenal azotemia. Early on, studies of biomarkers concentrated on using them as a tool to identify patients who had tubular injury and, thus, should be excluded from vasopressive therapies. By incorporating biomarkers along with clinical judgment and other arguments, clinicians can refine their diagnosis. One study combined uNGAL, IL-18, L-FABP, and albumin, defined specific cut-offs for each biomarker, and showed that patients with all biomarkers above the cut-off had a 91% of having ATN vs. 7% for those without any positive marker [48]. Later, the focus shifted more towards the ability to identify patients who had HRS and should benefit from specific therapies, even predicting which patients could benefit further from such therapy. In this context, a study included one hundred sixty-two patients with cirrhosis and acute kidney injury, followed until death, liver transplant, or 90 days after inclusion; 39.5% of these patients had hepatorenal syndrome. Notably, uNGAL was measured specifically in patients that did not improve after 48 h of initial management diagnosed according to the criteria of the International Club of Ascites. Even though levels of uNGAL were higher in patients presenting more severe AKI, they remained significantly increased in patients with ATN compared to patients with HRS regardless of AKI stage. A uNGAL level of 220 ng/mL was the best-performing threshold, with 89% of sensitivity and 78% of specificity. More interestingly, in the subgroup of patients presenting with HRS, those who responded completely to treatment with Terlipressin and albumin had lower levels of uNGAL compared to patients with partial or no response. Furthermore, uNGAL was an independent predictor of in-hospital and 90-day mortality [61].

IL-18 was also studied in this context but was found to have lower accuracy in predicting ATN vs. HRS but was associated with higher hospital mortality [53,53,62]. Using these biomarkers can also provide an additional argument in the decision between combined kidney–liver or liver transplantation alone [63].

Cardiorenal syndrome is another clinical scenario where decisions are not as straightforward, and both cardiologists and nephrologists often lack specific arguments to balance the need for diuretics and the rise in creatinine. Current therapeutic approaches involve diuretics to restore effective renal perfusion pressure and increase hemoconcentration, which are associated with decreased mortality and heart failure rehospitalization, even in the context of in-hospital worsening renal function [64]. As relying solely on clinical parameters may be insufficient to identify response to therapy, biomarkers can serve as an additional factor in this situation, as well as in resolving heterogeneity in pathophysiological mechanisms of cardiorenal syndromes. Urinary NGAL and IL-18 were found to be independently associated with AKI progression in patients with acute decompensated heart failure, and the addition of these biomarkers along with urinary angiotensinogen to a clinical model improved the risk stratification and identified the population with the highest risk of adverse kidney outcomes [54]. Baseline urinary L-FABP levels in patients hospitalized with acute decompensated heart failure were also found to be an independent predictor of AKI in these patients [47]. One paradigm was proposed by Parikh et al., where low levels of NGAL, N-acetyl-b-D-glucosaminidase, and KIM-1 support the continuation of diuretic therapy despite the rise in SCr [43]. While many biomarkers have been studied in this setting and shown to correlate well with heart failure hospitalizations, AKI, and mortality risk, there is still much work to be done in comparing these biomarkers and drawing direct consequences to facilitate their use in routine clinical decision making.

### 3.3. Diagnosing Acute Interstitial Nephritis

Diagnosis of acute interstitial nephritis (AIN) and distinction with acute tubular necrosis and other acute renal conditions can be challenging. It is crucial to establish the diagnosis of AIN in a timely manner since efficient therapeutics exist, entailing discontinuation of the causative agent or treating the underlying disease. The use of biomarkers in this context is also beneficial, providing an alternative to kidney biopsy in cases when diagnosis is unclear. To identify potential biomarkers, a study included 218 patients who underwent kidney biopsy for acute kidney injury from two different centers. The histological diagnosis of AIN was found in 15% of the biopsies, reflecting its actual proportion among the causes of acute kidney injury. Among twelve urine and ten plasma biomarkers measured, urinary TNF-α and IL-9 were independently associated with AIN, and their levels were higher in patients whose biopsies showed more severe histological lesions of AIN, such as tubulitis or lymphocytic and eosinophil infiltrates. What is noteworthy is the creation of two models: one comparing these biomarkers to a clinician’s judgment and the other comparing them to a model consisting of clinical variables typically associated with AIN. The addition of these biomarkers to both models significantly improved their ability to predict the diagnosis, raising the AUC of the clinicians from 0.62 to 0.84 and the clinical model from 0.69 to 0.84. This serves as an example of how biomarkers can be incorporated into clinical or other biological models, thereby enhancing their utility [65]. In addition, CXCL9 has recently been identified as a biomarker for AIN. It emerged as the top protein biomarker among 180 urine proteins associated with AIN in urine proteomics analysis of 88 patients with AKI, of which 35% had biopsy-proven AIN. Urinary CXCL9 also exhibited a correlation with the severity of histological lesions. This aligns with the fact that CXCL9 is a chemokine, by binding to CXCR-3, whose expression is induced by IFN-γ, guides activated T lymphocytes to the site of inflammation, primarily in tubular regions. The inclusion of CXCL9 in the previously mentioned models further enhanced their performance. Finally, the combination of CXCL9 with TNF-α and IL-9 was found to have the most diagnostic accuracy [66]. Tubulointerstitial nephritis with uveitis syndrome is another example of a tubulointerstitial disease that perfectly embodies a clinical context where a biomarker was incorporated in the classification criteria and can even help the clinician avoid a kidney biopsy. Evidence of tubulointerstitial nephritis can be proven either on kidney biopsy or in the presence of elevated urinary β_2_-microglobulin and either abnormal urine analysis or elevated serum creatinine [67].

### 3.4. Prediction of Contrast-Associated AKI

Many studies have suggested the potential use of biomarkers in predicting contrast-associated AKI, such as NGAL and IL-18 [40,68]. However, most were single-center studies with low event rates. Identifying high-risk patients can guide the nephrologist in approving the use of a contrast test, especially in non-urgent situations. It can also assist in determining which patients require hospitalization and close follow-up. In this regard, a larger study based on the PRESERVE trial cohort was conducted, measuring plasma biomarkers in 916 patients and urine biomarkers in 797 patients with chronic kidney disease: MCP-1, KIM-1, NGAL, IL-18, UMOD, and YKL-40 in 19 different centers. These markers were measured one to two hours pre-angiography. Patients with pre-angioplasty higher plasma KIM-1, NGAL, and YKL-40 were found to have a higher risk for major adverse kidney events such as the need for dialysis or persistent decrease in kidney function (defined as an increase in serum creatinine by >50%) as well as and death within 90 days (MAKE-D). Urine creatinine-corrected IL-18, MCP-1, and YKL-40 were also correlated with higher MAKE-D. Furthermore, plasma KIM-1 was significantly higher in patients presenting contrast-associated AKI, defined as an increase in serum creatinine of ≥25% or ≥0.5 mg/dL from baseline at 3-5 days after the injection [55].

### 3.5. AKI Biomarkers in Kidney Transplantation

Biomarkers also play a role in the field of kidney transplantation, where they could assist in decisions regarding donor selection and kidney graft monitoring. They can also have the potential to improve the prediction of graft survival risk, as demonstrated recently in a study involving 709 stable kidney transplant recipients. Urinary and plasma levels of NGAL and calprotectin were measured at least two months post-transplant, with a follow-up period extending to 58 months. The study revealed that plasma NGAL independently predicted renal allograft loss [69].

In the post-transplant context, another valuable tool for assessing kidney allografts is donor-derived cell-free DNA (dd-cfDNA), as it is released into the bloodstream with a half-life ranging from 30 to 120 min in the context of graft injury [70]. What appears clinically relevant is its high negative predictive value, helping clinicians avoid invasive biopsies in high-risk patients. In a retrospective observational study of 317 kidney transplant recipients with preserved kidney graft function, rejection episodes were more commonly found in patients with high dd-cfDNA (≥1%) compared to those with low dd-cfDNA (<0.5%) [71]. Furthermore, dd-cfDNA elevation precedes other clinical manifestations of rejection and detection of de novo donor-specific antibodies by several weeks. dd-cfDNA correlates strongly with antibody- and T-cell-mediated rejection, but it is not entirely specific to graft rejection as elevated levels are also observed in conditions such as acute tubular necrosis, pyelonephritis, and BK virus nephropathy [72].

Additionally, urinary CXCL9 and CXCL10 were found to effectively predict graft rejection when integrated into a clinical model with eGFR, donor-specific antibodies, and polyoma viremia. This integration led to a significant reduction in the number of protocol biopsies, specifically averting 59 protocol biopsies per 100 patients when the risk for rejection was predicted to be below 10% [56]. What differentiates these biomarkers from other noninvasive markers, such as cell-free DNA or mRNA markers, lies in the accessibility of the measurement technique and its simplicity. On the other hand, a recent meta-analysis revealed that biomarker studies in kidney transplantation lack validation, rigorous design, and comparison to standard-of-care graft monitoring, therefore highlighting the need for more efforts in the field [73].

## 4. Beyond the Horizon: Broadening Prognostic Perspectives

AKI is a well-established risk factor for the progression of chronic kidney disease (CKD) via pro-fibrotic pathways [74], and both renal syndromes are even considered as a continuum of the same deleterious process that constitutes a risk factor for cardiovascular diseases [75]. The role of biomarkers in understanding this continuum was addressed in a prospective cohort study involving 656 hospitalized AKI patients in which multiple urine and plasma biomarkers related to kidney injury, inflammation, and tubular health were assessed at various time points up to 12 months post-AKI. The authors observed that sustained tissue injury and inflammation, along with a slower restoration of tubular health, were associated with a higher risk of chronic kidney disease (CKD) incidence and progression. Specifically, increases in urine KIM-1, MCP-1, and plasma TNFR1 from baseline to 12 months correlated with a 2- to 3-fold increased risk of CKD, while an increase in urine uromodulin was associated with a 40% reduced risk [44]. 

Biomarkers may also play a role in predicting cardiovascular disease and mortality. A group of 502 cases were randomly subsampled from the Health ABC cohort, which includes well-functioning individuals aged between 70 and 79. Additionally, they enrolled 245 cases with cardiovascular disease and 220 cases with heart failure. A total of 776 individuals underwent a single time point urine measurement for α1m, NGAL, and amino-terminal propeptide of type III procollagen (PIIINP). The authors found that α1m and NGAL were independently associated with cardiovascular disease and mortality but not heart failure [76]. A similar association was also proved in the SPRINT cohort, with a sample of 2377 patients known to have CKD. Three urine biomarkers were measured: alpha-1 microglobulin (α1m), beta-2 microglobulin (β2m), and uromodulin. α1m was found to be associated with a higher risk of CVD and mortality, while uromodulin was associated with lower CVD risk [50]. Thus, several AKI biomarkers may be relevant to predict the risk of progression to CKD and subsequent cardiovascular events post-AKI.

## 5. Bridging the Gap: Linking Clinical Studies to Risk Stratification

AKI biomarkers are currently featured in several clinical trials as biological metrics intended to modulate clinical intervention and/or measure clinical outcomes such as risk of progression to CKD or mortality [77]. Indeed, AKI biomarkers can play a role in the early identification of patients at risk and in evaluating specific clinical management and therapeutic interventions at various stages of AKI. These biomarker-directed approaches are actively tested to implement the KDIGO care bundle. In the PrevAKI study, a single-center randomized trial, the efficacy of KDIGO guidelines in preventing cardiac surgery-associated AKI was evaluated in high-risk patients identified through renal biomarkers. This allowed for interventions to commence before the change in kidney function and, therefore, the onset of kidney damage. A total of 276 high-risk patients, defined as having a urinary [TIMP-2] × [IGFBP-7] > 0.3, were included. They were then divided into a control group receiving standard care and an intervention group strictly following the KDIGO CT surgery bundle. The primary outcome, consisting of the occurrence of AKI in the 72 h after surgery, was significantly lower in the intervention group (55.1 vs. 71.7%; ARR 16.6%, 95% CI 5.5–27.9; *p* = 0.004) [78]. A similar approach using the same biomarkers was examined in the BigpAK trial to assess the impact of the KDIGO care bundle in preventing AKI after noncardiac major surgery. Although overall AKI rates did not differ, episodes of moderate and severe AKI, ICU length, and hospital stay were significantly lower in the controlled group [79]. This study led to the initiation of the BigpAK-2, an international multi-center prospective randomized controlled trial expected to enroll 1302 patients to retest the primary outcome in a broader population [80].

In 2019, the Acute Disease Quality Initiative (ADQI) proposed recommendations on AKI biomarkers, focusing on the assessment, prediction, prevention, management, and progression of AKI. ADQI emphasized the importance of combining damage and functional biomarkers with clinical information to enhance the diagnostic accuracy of AKI, providing a deeper understanding of the pathophysiological processes that discriminate etiologies. Additionally, they proposed a new AKI classification by subdividing KDIGO stage 1 AKI into three subclasses: stage 1S, where kidney injury is detected only by a biomarker and is not evident through creatinine and urine output. Patients who meet the classic stage 1 criteria are then differentiated based on whether they have negative biomarkers in stage 1A or positive biomarkers in 1B. Furthermore, stages 2 and 3 were also subcategorized into A and B depending on the status of biomarkers [81]. During its controversies conference in 2019, KDIGO highlighted the emergence of biomarkers as one of the new tools for diagnosing and managing AKI. However, they considered that there is currently insufficient evidence to incorporate these biomarkers into the existing AKI definition. Furthermore, the limitation of their global availability makes this task even more challenging [82]. Despite their apparent practicality when accessible, the question of the cost-effectiveness of these tests remains unclear.

The economic evaluation of Nephrocheck, cystatin C, and NGAL was extensively studied in the context of early AKI detection in 2018. This revealed that, individually assessed against standard care, all tests seemed cost-effective. Sensitivity analyses highlighted Nephrocheck’s sensitivity to parameter changes, emphasizing the need for further research and comparisons with previous economic evaluations, underscoring the uncertainty and variability in outcomes, and stressing the importance of refining these analyses. Furthermore, generalizing these results beyond the ICU setting or the UK NHS, where the study was conducted, should be approached with caution [83].

This uncertainty was highlighted in a systematic review and cost-effectiveness analysis conducted to assess the role of biomarkers, including Nephrocheck, ARCHITECT urine NGAL, and BioPorto NGAL, in early AKI detection. The comprehensive review, encompassing 56 studies with 17,967 participants, revealed potential benefits in detecting and predicting AKI. However, notable limitations, such as a lack of studies addressing the clinical impact of biomarkers compared to standard care and uncertainties in optimal thresholds for NGAL, contribute to the overall uncertainty. This emphasizes the need for additional research to comprehensively assess the role and economic value of these biomarkers [84].

Finally, it is noteworthy to mention the rapid evolution in this field in parallel with the advancement of techniques and the emergence of other biomarker models. New research areas are employing proteomics to identify early urine peptide signatures in AKI [85]. Extracellular vehicles [86] and microRNAs [87] are also being studied as biomarkers and potential therapeutic targets for intervention. Ultimately, AKI biomarker assessment in combination and integrated with relevant clinical information should enable tailoring of AKI management and treatment of individual patients at early stages, with therefore the potential to improve clinical practice, save costs, and reduce risk of progression to CKD.

## Figures and Tables

**Table 1:** Summary of biological characteristics, clinical applications, and prognostic use of AKI biomarkers.

Biomarker	Sample Type	Secretion Site	Pathological Process	Applications	Prognostic Use	Score Integration	References
NGAL	Urinary NGAL Plasma NGAL	uNGAL: thick ascending limb of the loop of Helen, collecting ducts pNGAL: secreted in various organs in response to inflammatory signals, freely filtered, then completely reabsorbed via the proximal tubule	Tubular injury (proximal)	uNGAL: prediction of AKI and mortality after cardiac surgery pNGAL: prediction of AKI and mortality after cardiac surgery Prediction of contrast-induced MAKE-D Prediction of renal allograft loss	Progression to CKD Predicts mortality but also response to therapy in HRS Cardiovascular disease	Part of the CSA-NGAL score for risk stratification in cardiac surgery Was used in combination with other biomarkers (IL-18 and L-FABP) in the prediction of HRS	[8,11,38,39,40,41]
KIM-1	Urinary KIM-1 Plasma KIM-1	Proximal tubule	Tubular injury (proximal)	uKIM-1: prediction of AKI and mortality after cardiac surgery pKIM-1: Prediction of contrast-induced MAKE-D	Progression to CKD		[11,42,43]
TIMP-2	Urine	Distal convoluted tubule Collecting duct	Tubular injury; cell cycle arrest	Prediction of AKI after major cardiac surgery		NEPHROCHECK	[13,14,44]
IGFBP-7	Urine	Distal convoluted tubule Collecting duct Proximal tubule Podocytes	Tubular injury; cell cycle arrest	Prediction of AKI after major cardiac surgery		NEPHROCHECK	[14,15,44]
α1-M	Urine	Proximal tubule	Tubular function	Prediction of AKI and mortality after major cardiac surgery	Prediction of cardiovascular disease and long-term mortality		[18,45,46]
L-FABP	Urine	Proximal tubule	Tubular function	Prediction of AKI and mortality after major cardiac surgery Prediction of AKI in cardiorenal syndrome	Prognostic marker for increased mortality and risk of acute-on-chronic liver failure development	Used in combination with other biomarkers (IL-18 and NGAL) in the prediction of HRS	[21,47,48,49]
UMOD	Urine	Thick ascending limb of the loop of Helen	Tubular function	Lower levels have been linked to a higher risk of postoperative AKI in cardiac surgery	Lower-risk cardiovascular disease and CKD progression		[22,44,50,51]
IL-18	Urine		Kidney inflammation	Prediction of AKI and mortality after major cardiac surgery Prediction of AKI in cardiorenal syndrome Prediction of contrast-induced MAKE-D	Prognostic marker for in-hospital mortality for cirrhotic patients with AKI	Used in combination with other biomarkers (L-FABP and NGAL) in the prediction of HRS	[48,52,53,54,55]
MCP-1	Urine	Podocytes	Kidney inflammation	Prediction of AKI and mortality after major cardiac surgery Reflects endothelial dysfunction and activity in lupus nephritis. Prediction of contrast-induced MAKE-D	Progression to CKD		[31,32,55,56,57]

**Table 2:** Overview of ongoing clinical trials, encompassing both observational and interventional studies focused on or utilizing biomarkers.

Trial ID	Name	Country	Study Design	Biomarker	Question	Estimated Enrollment
NCT05907434	Cell Cycle Arrest Proteins for Early Diagnosis of Acute Kidney Injury After Lung Transplant (LUTX_AKI)	Italy	Observational	urinary [TIMP-2] × [IGFBP-7]	Employing urinary [TIMP-2] × [IGFBP-7] measured on postoperative day as an early predictor of AKI following lung transplant	40
NCT05283213	Development of an Innovative Clinico-biological Score for the Early Detection of Acute Renal Failure Associated With Cardiac Surgery. (DETECT-AKI)	France	Observational	Urinary TIMP-2 Plasma NGAL Urinary NGAL Plasma cystatin C Plasma HI Plasma IL-6 Urinary DKK3 Creatinine Uremia	Develop a composite score from clinical and biological data to guide decision making after cardiac surgery, followed by an internal validation cohort	400
NCT04647396	Biomarker- Guided Intervention to Prevent Acute Kidney Injury After Major Surgery. A Prospective Randomized Controlled Multicenter Trial (BigpAK-2)	Belgium, France, Germany, Italy, Netherlands, Spain, Switzerland, United Kingdom	Interventional	urinary [TIMP-2] × [IGFBP-7]	Study the effect of the implementation of the KDIGO bundle in patients with a high risk of AKI after cardiac surgery with a biomarker-guided approach	1302
NCT06124885	Real-time Early Detection of Nephrotoxicity by Accurate and Faster Urinary Biomarker Analysis With SeroFlow Technology (RenaFAST Study)	Singapore	Observational	Clusterin MCP-1 B2MG	Validation of the RnaFAST kit in predicting AKI	150
NCT05285709	Evaluation of Novel Biomarkers in Early Recognition of Acute Kidney Injury After Orthopedic Operations.	Poland	Observational	PENK DKK3 circRNA L-FABP Netrin-1 Semaphorine-3 TIMP-2 IGFBP-7 RBP-4	The role of biomarkers in predicting early AKI after orthopedic surgery	200
NCT03263325	Evaluation of UDP-glucose as a Urinary Biomarker for Early Detection of Cardiac Surgery-associated Pediatric Acute Kidney Injury	USA	Observational	UDP-glucose	The role of urinary UDP-glucose in predicting AKI in children ≤ 8 years of age undergoing cardiac surgery	250
NCT05349292	Effect of Low-volume Fluid Replacement Strategy During Acute Normovolemic Hemodilution on Urine Neutrophil Gelatinase-associated Lipocalin Levels: an Acute Kidney InjuryBiomarker	USA	Observational	Urinary NGAL Urinary KIM-1	Determining whether there is an increased risk for kidney injury with the use of ANH based on biomarkers	100
NCT05275218	Effect of an Extended KDIGO Bundle Versus Standard of Care Therapy on Persistent Acute Kidney Injury in High-risk Patients After Major Surgery	Germany	Interventional	CLL14	Study the effect of the KDIGO bundle after a major surgery while stratifying the patients into risk categories based on the levels of CCL14	480
NCT02375854	Outcomes of Neonatal Acute Kidney Injury and Prematurity: An Observational Study and Einstein-Montefiore Neonatal Acute Kidney Injury Clinical Registry and Biorepository	USA	Observational	Serum cystatin C Urinary NGAL Urinary IL-18 Urinary KIM-1	Five-year follow-up of neonatal AKI, with biomarkers as one of the outcome measures	200
NCT04408248	AKI Biomarkers for Prediction of Acute Kidney Injury in Critically Ill Patients With COVID-19 and Respiratory Disease	United Kingdom	Observational	urinary [TIMP-2] urinary [IGFBP-7]	Early prediction of AKI in critically ill COVID-19 patients based on biomarkers	30
NCT05830669	Effect of Remote Ischemic Preconditioning in Septic Patients on Cell Cycle Arrest Biomarkers—the RIPC-ICU Randomized Clinical Trial	Germany	Interventional	urinary [TIMP-2] × [IGFBP-7]	Evaluate the effects of remote ischemic conditioning, identified by urinary biomarkers in critically ill patients on acute kidney injury	64
NCT04705766	The KIDCOV Study: Assessment of SARS-CoV-2 Without Hospitalisation as a risk factor for acute kidney injury	USA	Observational	Urinary NGAL Urinary KIM-1 Plasma suPAR	Using biomarkers as an outcome measure for evaluating AKI in COVID-19 patients	2000

## Data Availability

No new data were generated for this review. All data and sources cited in this article are publicly available through the original references provided.
